# Effect of Nb/C Ratio on Microstructure and Mechanical Properties of B50A789G Precipitation Hardening Stainless Steel

**DOI:** 10.3390/ma18091917

**Published:** 2025-04-23

**Authors:** Shuai Liu, Jiqing Zhao, Ruishan Xin, Yudong He, Gang Yang, Bin Yang

**Affiliations:** 1 Collaborative Innovation Center of Steel Technology, University of Science and Technology Beijing, Beijing 100083, China; shuailiu0809@126.com; 2 Special Steel Stitute, Central Iron & Steel Research Institute, Beijing 100081, China; yanggang@cisri.com.cn; 3 Ansteel Beijing Research Institute Co., Ltd., Beijing 102209, China; ruishanxin@163.com; 4 Jiangyou Changcheng Special Steel Co., Ltd. of Pangang Group, Jiangyou 621704, China; yudong9508@163.com

**Keywords:** B50A789G stainless steel, Nb/C ratio, microstructure, mechanical properties, strengthening mechanism

## Abstract

In this study, the microstructural evolution and mechanical properties of B50A789G steels with different Nb/C ratios (7, 9, 11, and 13) after tempering at 495 °C were investigated through mechanical property testing, X-ray diffraction (XRD), scanning electron microscope (SEM), and transmission electron microscope (TEM). The role of Nb in B50A789G steel was also explored. The results indicate that, at the same tempering time, the strength and hardness of the steel increase with increasing the Nb/C ratio. The maximum tensile strength exceeded 1240 MPa when the Nb/C ratio reached 13. With prolonged tempering time, the tensile strength of steels with low Nb/C ratios (7 and 9) gradually decreases, whereas steels with high Nb/C ratios (11 and 13) exhibit a decline in tensile strength only after 6 h of tempering. In contrast, the impact toughness shows an opposite trend to the strength. As the Nb/C ratio increases, both coarse primary NbC and nanoscale NbC precipitates in the steel gradually increase. The primary roles of Nb in B50A789G steel are grain refinement strengthening and precipitation strengthening. For steels with Nb/C ≤ 11, the improvement in strength is attributed to the combined effects of grain refinement strengthening and precipitation strengthening provided by Nb. However, for steels with Nb/C > 11, the increase in strength is primarily driven by the precipitation-strengthening effect of the nanoscale NbC phase.

## 1. Introduction

Precipitation-hardened stainless steels are characterized by their high strength [1], excellent toughness [2], superior corrosion resistance [3], and remarkable resistance to water droplet erosion [4], coupled with relatively low complexity in both hot and cold working processes [5,6,7,8]. These advantageous properties have established them as the material of choice for the fabrication of last-stage long blades in steam turbines. Currently, leading manufacturers such as Mitsubishi, General Electric Company (GE), and Siemens have widely adopted these steels for this critical application [9,10,11,12,13].

Precipitation-hardened stainless steels can be classified into two categories based on their primary strengthening mechanisms: steels strengthened predominantly by the precipitation of ε-Cu phase and carbides (17-4PH and GTD-450) [14,15], and steels strengthened primarily by the β-NiAl phase (PH13-8Mo) [16,17,18]. The GTD-450 steel, designated as B50A789G under specification of GE, was initially developed for compressor blades. Its strengthening mechanism is analogous to that of 17-4PH steel, involving the formation of low-carbon martensite upon rapidly cooling from the solution temperature to room temperature [19], followed by the precipitation of strengthening phases (carbides and Cu-rich phases) within the martensitic matrix during aging treatment, thereby enhancing the matrix strength [20,21]. The carbide responsible for precipitation strengthening in B50A789G steel is NbC. Studies reported in the literature [22,23,24] indicate that NbC phases precipitate during various thermal processes, including hot forming, normalizing, tempering, and prolonged high-temperature aging. The NbC phases that precipitate during the tempering process are finely dispersed within the martensitic matrix. Due to their slow coarsening rate, these phases significantly contribute to maintaining the strength and toughness of the material [25]. Liu et al. [26] found that the presence of high-density nano-NbC particles in tempered martensite can maintain a high yield strength, and the hardness rarely decreases after long-term aging. Zhang et al. [22] indicated that by adding more Nb to steel, the dispersion and size of NbC precipitates become smaller, thereby achieving better strengthening effects.

As a crucial alloying element in precipitation-hardening stainless steels, Klinkenberg et al. [27] demonstrated that Niobium (Nb) provides more nuclei for the γ/α transformation and thus a finer grain size. However, B50A789G steel contains a relatively high niobium content, which is 8 to 15 times that of carbon. This high Nb/C ratio can lead to the formation of a large primary NbC phase, which may adversely affect the performance of the material. Zhou et al. [28] indicated that as the Nb content in the steel increases, the quantity, particle size, and initial precipitation temperature of niobium carbides (NbC) precipitated in the matrix structure all increase. The primary NbC precipitates can be effectively analyzed through thermodynamic calculations to elucidate their role in steel systems [29,30]. Tao et al. [31] investigated the influence of NbC on austenite grain size at elevated temperatures through simulation and TEM characterization. Their results revealed that NbC particles larger than the average size not only failed to inhibit austenite grain growth, but also induced abnormal grain growth.

Current research predominantly focuses on qualitative investigations of the role of niobium in precipitation-hardening stainless steels, while quantitative studies elucidating the contribution of NbC precipitation to the actual strength of steels remain limited. Furthermore, the correlation between niobium content and its mechanistic role in stainless steels has not been clearly established. In this context, the present study systematically investigates the microstructural evolution and mechanical properties of B50A789G steels with varying Nb/C ratios, with particular emphasis on elucidating the relationship between niobium content and its underlying mechanisms in steel.

## 2. Experimental

### 2.1. Material

In this study, four steels were designed with carbon content controlled at the middle of the content range. By varying the niobium content, steels with different Nb/C ratios were produced. The steels were melted using a 25 kg vacuum induction furnace and subsequently forged into rods with a diameter of 18 mm. The actual chemical compositions of the steels are provided in Table 1, with measured Nb/C ratios of 7, 9, 11, and 13, respectively. According to the technical specifications, the steels with varying Nb/C ratios were subjected to heat treatment. The heat treatment regimen consisted of the following steps: holding at 1038 °C for 1 h followed by oil quenching, and then holding at 495 °C for 2, 4, 6, and 8 h, respectively, followed by air cooling.

### 2.2. Mechanical Properties Testing

Mechanical property testing was conducted to evaluate the performance of the steels under various conditions. The tests included tensile strength, yield strength, hardness, and impact toughness measurements. After heat treatment, the specimens were machined into standard tensile test samples with dimensions of M12 × φ5. Tensile tests were conducted on a WE-300 testing machine (HST Group, Dongguan, China) in accordance with the GB/T 228.1 [32]. For impact testing, V-notch specimens measuring 10 mm × 10 mm × 55 mm were prepared and tested on a JBN-300B impact testing machine (SANS, Shenzhen, China) following the GB/T 229 [33]. Additionally, hardness measurements were performed on the impact test specimens using an HB3000C electronic hardness tester (HST Group, Dongguan, China).

### 2.3. Microstructure Characterization

The specimens were sectioned, ground, and polished, followed by etching in a mixed solution of CuCl_2_ + FeCl_3_ + H_2_O + HNO_3_ + C_2_H_5_OH for 10–15 s. The microstructure of the etched surface was observed using a scanning electron microscope (SEM, Quanta 650FEG, FEI, Hillsboro, OR, USA). Samples with a thickness of 0.5 mm were prepared by grinding on water-lubricated sandpaper to achieve 0.05 mm thin foils, which were subsequently cut into 3 mm diameter discs. Electrolytic double-jet thinning was performed using a 10% perchloric acid–alcohol solution at a constant current of 50 mA, with a perforation size of 50, and low-temperature thinning at −20 °C. The prepared samples were examined under a transmission electron microscope (TEM, JEM-2100F, JEOL, Tokyo, Japan) to observe the precipitation of fine NbC, as well as the microstructure. Diffraction pattern analysis was conducted using Gatan Digital Micrograph software (https://www.gatan.com/products/tem-analysis/gatan-microscopy-suite-software), and the size distribution of secondary phase particles was statistically analyzed using Nano Measurer software (https://nano-measurer.software.informer.com/). The X-ray diffraction (XRD D/max-2400 X-ray diffractometer, Rigaku, Tokyo, Japan) test was conducted using the following experimental method: After electrolytic extraction of the steel samples, the residues were subjected to small-angle diffraction analysis. The diffraction angle (2θ) range was set from 20° to 10°, with a scanning step size of 0.02°. A Cu target was used for the analysis to identify the precipitates in the steel.

## 3. Results

### 3.1. Effect of Nb/C Ratio on Microstructure

**Figure 1:**
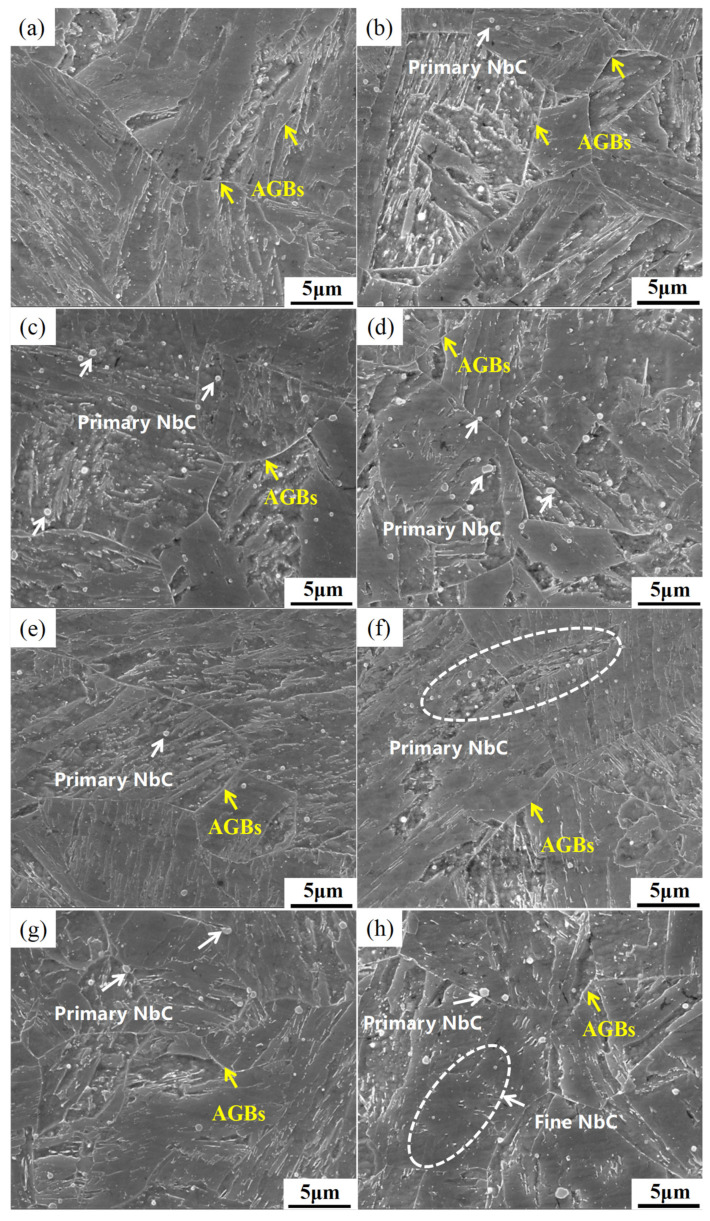
SEM images of steel under different tempering time: (**a**) Nb/C = 7 tempered for 4 h; (**b**) Nb/C = 9 tempered for 4 h; (**c**) Nb/C = 11 tempered for 4 h; (**d**) Nb/C = 13 tempered for 4 h; (**e**) Nb/C = 7 tempered for 6 h; (**f**) Nb/C = 9 tempered for 6 h; (**g**) Nb/C = 11 tempered for 6 h; (**h**) Nb/C = 13 tempered for 6 h.

Figure 1 presents the SEM microstructures of four steels tempered at 495 °C for varying durations. As illustrated in Figure 1, with increasing Nb/C ratio, both the quantity and size of large primary NbC phases in the steel increase, distributed along prior austenite grain boundaries and within grains. Simultaneously, the number of fine precipitates within grains gradually increases with higher Nb/C ratios. Comparative analysis of the four steels with different Nb/C ratios reveals that the decomposition of lath structures is delayed with increasing Nb/C ratio. Specifically, in the steel with Nb/C = 7, lath decomposition is observed after 4 h of tempering at 495 °C, whereas the steel with Nb/C = 13 retains partial lath morphology under the same tempering conditions. This indicates that under the same tempering temperature and duration, an increase in NbC precipitation can more effectively pin martensitic laths, thereby providing higher strength to the material. When the tempering duration is extended from 4 to 6 h, the martensitic structure in all steels undergoes decomposition, accompanied by an increased number of fine NbC precipitates within grains.

**Figure 2:**
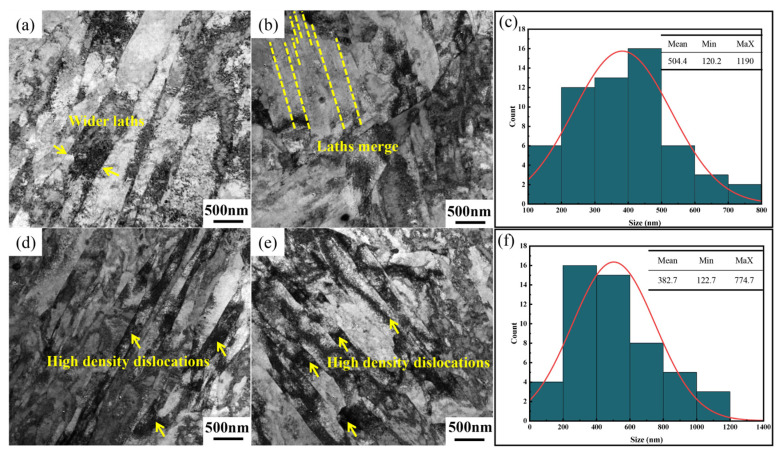
TEM images under different tempering time: (**a**) Nb/C = 9 tempered for 4 h; (**b**) Nb/C = 9 tempered for 6 h; (**c**) lath width of steel with Nb/C = 9 temped for 6 h (**d**) Nb/C = 13 tempered for 4 h; (**e**) Nb/C = 13 tempered for 6 h; (**f**) lath width of steel with Nb/C = 13 temped for 6 h.

To further investigate the evolution of martensitic laths under different Nb/C ratios, TEM observations were conducted on the steels, as shown in Figure 2. At 495 °C with 4 h of tempering, distinct dislocations between laths were observed in both Nb/C = 9 and Nb/C = 13 steels. However, the lath width in the Nb/C = 9 steel was greater than that in the Nb/C = 13 steel. After 6 h of tempering, the lath width in the Nb/C = 9 steel further increased, while the lath morphology in the Nb/C = 13 steel remained largely unchanged. Statistical analysis revealed that after 6 h of tempering, the average lath widths of the Nb/C = 9 and Nb/C = 13 steels were 504.4 nm (Figure 2c) and 382.7 nm (Figure 2f), respectively. These results further demonstrate that increasing the Nb/C ratio effectively retards the broadening of martensitic laths and delays the onset of lath decomposition at the same temperature.

### 3.2. Effect of Nb/C Ratio on NbC

**Figure 3:**
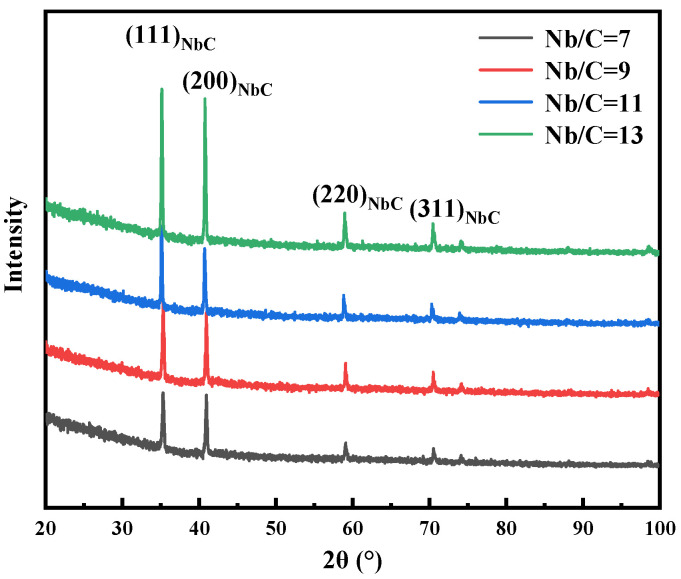
X-ray diffraction patterns of the carbides extracted from steel with different Nb/C after being tempered at 495 °C for 6 h.

Figure 3 presents the XRD patterns of precipitate particles in B50A789G steels with different Nb/C ratios after tempering at 495 °C for 6 h. Distinct NbC peaks corresponding to various crystallographic planes are observed in all steels, regardless of the Nb/C ratio. However, no peaks corresponding to Cu-rich phases are detected, likely due to the extremely small size of Cu-rich particles, which makes them difficult to resolve in the XRD patterns. As the Nb/C ratio increases, the intensity of the NbC peaks gradually rises, with a particularly significant increase observed in the steel with an Nb/C ratio of 13. This indicates that the precipitation of NbC in B50A789G steel is markedly enhanced when the Nb/C ratio reaches 13.

**Figure 4:**
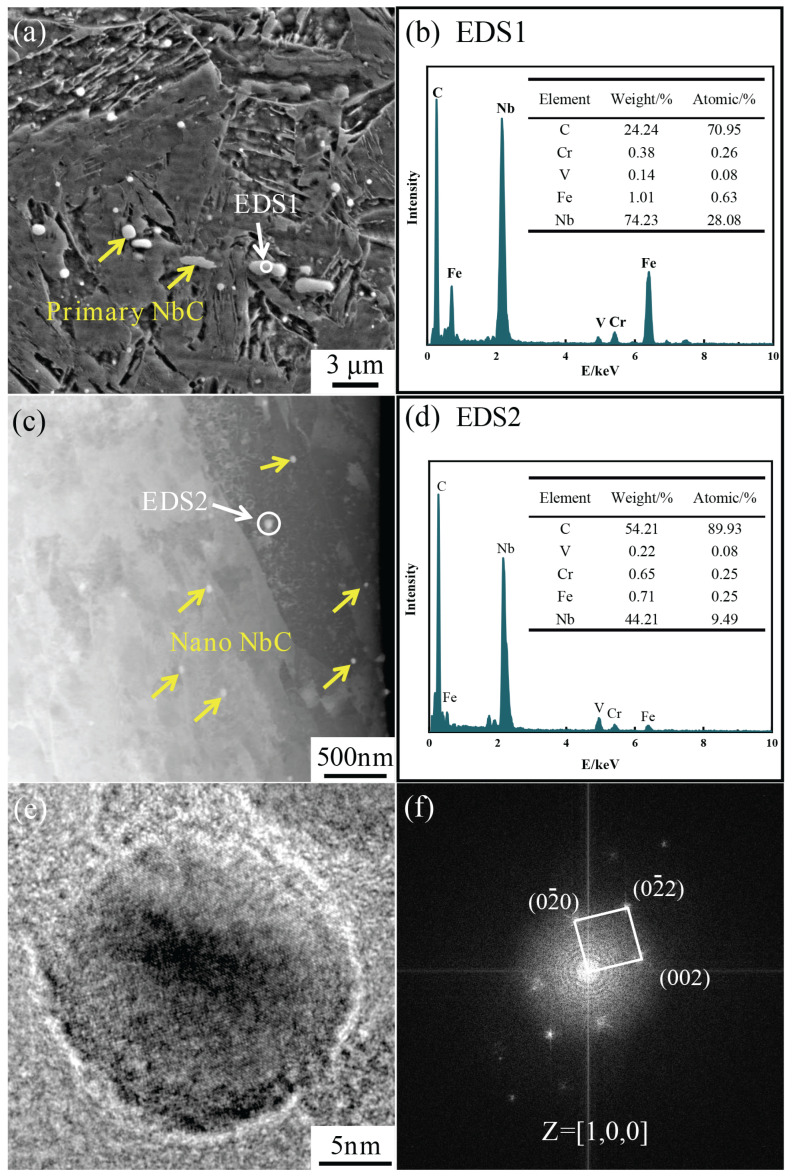
Microstructure of Nb/C = 9 steel after tempering at 495 °C for 6 h: (**a**) SEM image; (**b**) EDS1; (**c**) TEM dark-field image; (**d**) EDS2; (**e**) HRTEM image of NbC; (**f**) IFFT of (**e**).

Figure 4 illustrates the microstructural characteristics of B50A789G steel following tempering at 495 °C for 6 h. The SEM micrograph in Figure 4a reveals the morphology of primary NbC phases, with some exhibiting a short rod-like configuration. EDS analysis indicates that the Nb content in these primary phases reaches 74.23 wt% (Figure 4b). The dark-field image in Figure 4c demonstrates the precipitation of nano-sized NbC carbides both within the martensite laths and along their boundaries. Notably, EDS analysis reveals a distinct compositional difference between the nano-sized and primary NbC phases, with the former containing 44.21 wt% Nb. Figure 4e presents an HRTEM image of the NbC phase, with the corresponding IFFT shown in Figure 4f. The crystallographic characteristics of NbC were confirmed through systematic calibration of these images.

**Figure 5:**
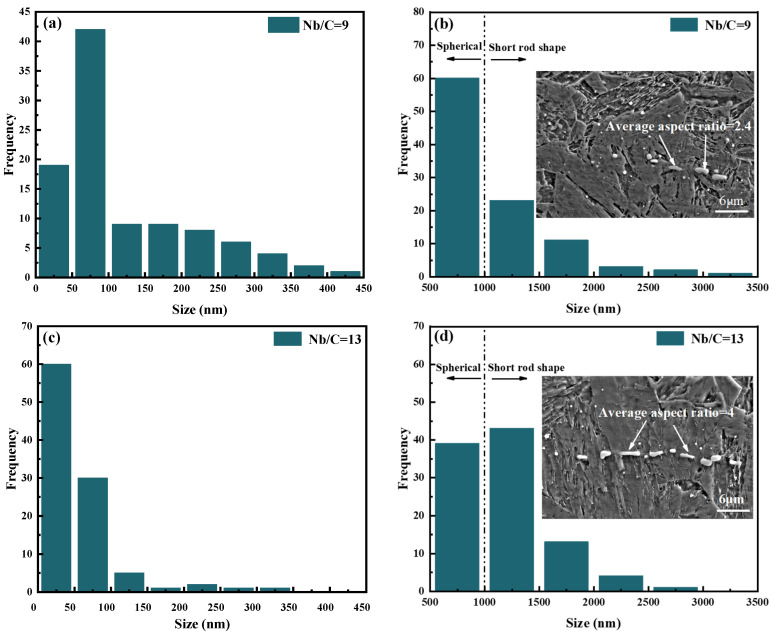
Statistics results of NbC phase size: (**a**) results from TEM images of Nb/C = 9 steel, (**b**) results from SEM images of Nb/C = 9 steel, (**c**) results from TEM images of Nb/C = 13 steels, (**d**) results from SEM images of Nb/C = 13 steels.

Figure 5 presents the size distribution of NbC precipitates in the steels with Nb/C ratios of 9 and 13. As shown in Figure 5a,c, the proportion of NbC precipitates smaller than 50 nm in the steel with an Nb/C = 13 is higher than that in the steel with an Nb/C = 9. This suggests that increasing the Nb/C ratio from 9 to 13 promotes the precipitation of nanoscale NbC. In both steels, NbC precipitates larger than 1 μm exhibit a short rod-like morphology with a directional alignment, which is attributed to the elongation of coarse primary NbC phases perpendicular to the forging direction during the forging process. As illustrated in Figure 5b,d, the proportion of primary NbC phases larger than 1 μm in the steel with an Nb/C ratio of 13 is significantly higher compared to that in the steel with an Nb/C = 9. Additionally, the aspect ratio of the rod-like primary NbC phases increases from 2.4 to 4. Under the same forging ratio, these changes indicate that the primary NbC phases in the steel with an Nb/C = 13 are larger in size.

### 3.3. Effect of Nb/C Ratio on Mechanical Properties

**Figure 6:**
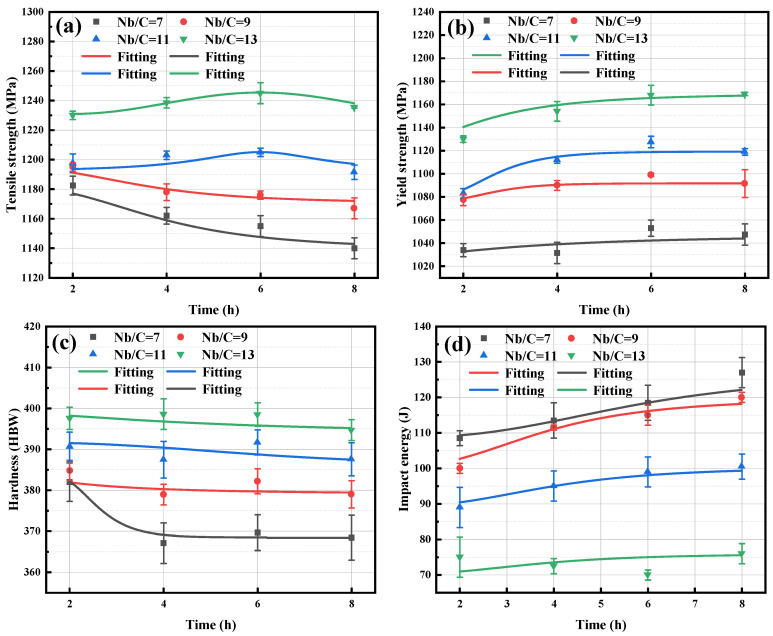
Effect of Nb/C ratio on mechanical properties of test steel under different tempering times: (**a**) tensile strength; (**b**) yield strength; (**c**) hardness; (**d**) impact energy.

Figure 6 presents the mechanical properties of the four steels tempered at 495 °C for different durations. With prolonged tempering time, the tensile strength of steels with Nb/C ratios of 7 and 9 gradually decreases. In contrast, for steels with Nb/C ratios of 11 and 13, the tensile strength initially increases slightly and then begins to decline after 6 h. The maximum tensile strength exceeded 1240 MPa when the Nb/C ratio reached 13. Regarding yield strength, steels with Nb/C ratios of 7, 9, and 11 start to decrease after 6 h of tempering, while the yield strength of the steel with an Nb/C ratio of 13 shows no declining trend even after 8 h. This is mainly attributed to the pinning effect of NbC on the matrix during aging treatment. With the increase of Nb/C ratio in steel, the amount of NbC precipitation increases, which can better prevent the decomposition of martensite laths and delay the strength decline time of steel with high Nb/C ratio (Nb/C = 9 and 13).

The hardness of the steels with different Nb/C ratios exhibits minimal change with prolonged tempering time at 495 °C, with variations within approximately 10 HB. In terms of impact energy, steels with Nb/C ratios of 7 to 11 show a gradual increase with extended tempering time, whereas steel with an Nb/C ratio of 13 initially decreases and only begins to rise after 6 h. The Nb/C ratio has a significant influence on impact energy: as the Nb/C ratio increases, the impact energy of the steels decreases, and for steels with Nb/C > 10 (Nb/C = 11 and 13), the impact energy shows a more pronounced reduction compared to steels with lower Nb/C ratios. This is due to the fact that the high Nb/C ratio leads to more precipitation of primary NbC phases in the steel, and the large size of the primary NbC is a brittle phase that is prone to becoming a crack source under impact loads, and cannot be cut by dislocation by the Orowan bypass mechanism, which promotes brittle fracture. These reasons have been confirmed in the study by Ji et al. [34].

## 4. Discussion

With prolonged tempering duration, the steel with a lower Nb/C ratio exhibits a discernible decreasing trend in strength. This is primarily attributed to the coarsening of precipitates over time, which reduces their pinning effect on dislocations. In B50A789G steel, the main strengthening phases are nanoscale Cu-rich precipitates and NbC precipitates. Since the actual Cu content in the four steels is similar, the strengthening contribution from the Cu-rich precipitates is nearly identical. Therefore, the differences in microstructure and mechanical properties among the steels are predominantly caused by the varying Nb content resulting from different Nb/C ratios.

### 4.1. Grain Refinement Strengthening Effect of Nb

Studies have shown [35,36,37,38,39,40] that the addition of a certain amount of Nb to steel can significantly refine the grain structure. On the one hand, solute Nb exerts a drag effect on grain boundaries, influencing the rate of boundary migration [41,42,43]. On the other hand, the presence of undissolved NbC phases at high temperatures, combined with the pinning effect of second-phase particles at austenitizing temperatures, reduces the tendency of austenite grain growth. According to the theory of solubility product for second-phase particles in steel materials, the commonly used solubility product formula for Nb in austenite is given by [44].

(1) $$\mathrm{lg}{([C]•[\mathrm{Nb}])}_{\gamma }=2.96-7510/T$$

(2) $$\frac{\mathrm{Nb}-[\mathrm{Nb}]}{C-[C]}=\frac{A_{\mathrm{Nb}}}{A_{C}}$$

where [C] and [Nb] represent the solid solubility of C and Nb in austenite, respectively; T is the thermodynamic temperature (the solution temperature used in this study is 1311 K); A_Nb_ is the atomic weight of Nb in NbC, with a value of 92.9064; and A_C_ is the atomic weight of C in NbC, with a value of 12.011. By combining Equations (1) and (2), the solid solubility of Nb in austenite at 1038 °C (1311 K) for steels with different Nb/C ratios was calculated, and the results are presented in Table 2. It can be observed that a significant portion of Nb exists in the form of second-phase particles in all steels at 1038 °C, regardless of the Nb/C ratio. For steels with Nb/C ≤ 11, the solid solubility of Nb gradually increases with the Nb/C ratio. However, for steels with Nb/C = 11 and 13, the solid solubility of Nb remains nearly identical.

In Nb-alloyed steels, the driving force required for grain boundary migration during grain growth is typically small. Under such conditions, the relationship between grain boundary mobility and solute concentration in the solute drag model can be simplified as [45]

M_eff_ = M_0_ (1 + M_0_ λ C_s_)^−1^ (3)

where M_eff_ is the effective grain boundary mobility, M_0_ is the intrinsic grain boundary mobility in the absence of solute atoms, λ is the interaction parameter representing the drag effect of solute atoms on the grain boundary, and C_s_ is the solid solubility of Nb. From Equation (3), it can be observed that the higher the solid solubility of Nb in the matrix, the lower the grain boundary mobility, indicating that the drag effect of solute Nb on grain boundaries increases with the Nb/C ratio. For the steels with Nb/C ratios of 11 and 13, since the solid solubility of Nb is nearly identical, the drag effect of solute Nb on grain boundaries is essentially the same in both steels.

The NbC phase can effectively inhibit grain growth only when its particle size is below a critical threshold. Once the NbC particle size exceeds this critical value, it is preferable to suppress NbC precipitation and maintain Nb in a solid solution state to maximize its grain-refining effect. In Figure 2, except for the steel with an Nb/C ratio of 7, large primary NbC particles were observed in the SEM microstructures of the other three steels. To quantify the amount of primary NbC precipitates in steels with different Nb/C ratios, equilibrium phase diagrams were calculated using Thermo-Calc software (https://thermocalc.com/). The amount of primary NbC precipitates was determined based on the temperature at which the steel transitions to a fully austenitic structure. The Nb content in these primary NbC precipitates was then calculated using Equation (2), and the results are summarized in Table 3. It indicated that the amount of primary NbC precipitates increases with the Nb/C ratio. For the steel with an Nb/C ratio of 7, the primary NbC phase remains fully dissolved at the full austenitizing temperature, which is consistent with the scarcity of large primary NbC particles observed in the SEM images (Figure 2a,e). In contrast, the steel with an Nb/C ratio of 13 exhibits a significant increase in the amount and size of primary NbC precipitates, indicating that a large portion of Nb forms coarse NbC particles, which are less effective in inhibiting grain growth. This suggests that the prior austenite grain size does not continuously decrease with increasing Nb content.

**Figure 7:**
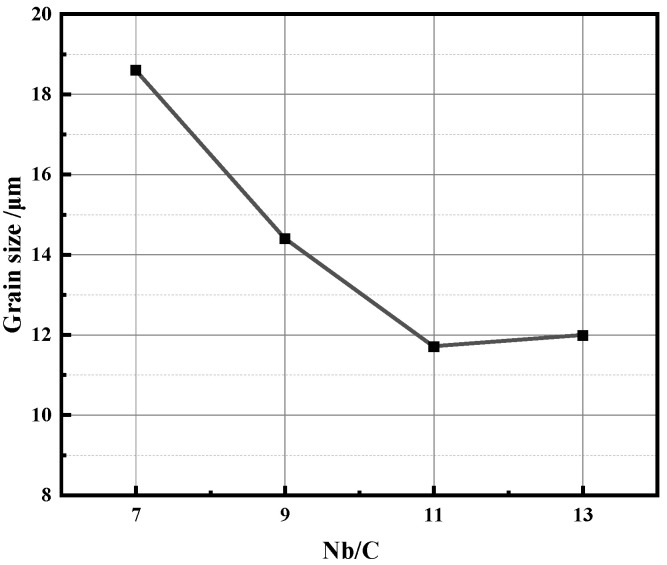
The prior austenite grain size of steel with different Nb/C.

Figure 7 presents the prior austenite grain sizes of the steels with different Nb/C ratios after treatment at 1038 °C quenching followed by 495 °C tempering for 6 h, as measured by the intercept method. As the Nb/C ratio increases from 7 to 11, the prior austenite grain size of the steels continuously decreases. Refined austenite grains lead to a reduction in martensitic lath/bundle or block size, an increase in lath boundary density, and enhanced dislocation motion obstruction, thereby improving strength. However, when the Nb/C ratio increases from 11 to 13, the grain size no longer decreases. This indicates that there is an upper threshold for the amount of Nb that can effectively refine the grain structure. Beyond this threshold, further increases in Nb content do not lead to additional grain refinement, and grain refinement strengthening ceases to be the primary contributor to the increase in strength.

### 4.2. Precipitation Strengthening Effect of NbC Phase

The NbC phases precipitated at high temperatures are generally coarse [46], and under impact loading, cracks tend to initiate around these large NbC particles, which is detrimental to the impact toughness of the material. In contrast, the NbC phases precipitated during the tempering process are fine [47], typically ranging from 2 to 20 nm, and are predominantly distributed at dislocations within the martensitic matrix [48]. These fine precipitates effectively hinder dislocation motion, contributing significantly to strengthening. The smaller the precipitate size and the larger the volume fraction, the more pronounced the precipitation strengthening effect [49]. By comparing the mechanical properties of B50A789G steel in Figure 6 with the grain size evolution in Figure 7, it is evident that when the Nb/C ratio increases from 11 to 13, the strength continues to rise while the grain size remains relatively unchanged. This suggests that grain refinement strengthening is no longer the primary factor driving the increase in strength. Figure 5 shows that as the Nb/C ratio increases, the proportion of fine NbC precipitates gradually rises, leading to enhanced precipitation strengthening. Therefore, for steels with Nb/C > 11, the improvement in strength is primarily attributed to the precipitation strengthening effect of fine NbC phase. In the steel with an Nb/C = 13, the higher proportion of fine NbC precipitates results in stronger dislocation pinning, leading to higher strength compared to steels with lower Nb/C ratios. However, the size and volume fraction of primary NbC phases also increase, which contributes to the relatively lower impact energy.

According to Gladman’s theory [50], the increment in yield strength can be described using the modified Ashby–Orowan model, as expressed by Equation (4):

(4) $$\Delta \sigma_{p}=\frac{0.538\mathrm{Gb}f^{1/2}}{D}\ln(\frac{D}{2b})$$

In the equation, $\Delta \sigma_{p}$ represents the increment in yield strength, G is the shear modulus, which is 81,600 MPa for the iron matrix; b is the magnitude of the Burgers vector, which is 0.248 nm for ferrite; *D* is the average diameter of the precipitate particles, selected based on the data in Figure 5 for NbC phases smaller than 20 nm; and ƒ is the volume fraction of the precipitate particles. The volume fraction of the precipitate particles can be calculated using Equation (5) [51]:

(5) $$f=\omega \frac{\rho_{\mathrm{Fe}}}{\rho_{\mathrm{Nb}(\mathrm{CN})}}$$

where *ω* is the mass fraction of NbC, $\rho_{\mathrm{Fe}}$ is the density of pure iron, taken as 7.875 g/cm^3^, and $\rho_{\mathrm{NbC}}$ is the density of NbC, taken as 7.803 g/cm^3^. Table 4 presents the mass fractions of elements in NbC precipitates, as determined by physicochemical phase analysis, for steels with varying Nb/C ratios after 6 h of tempering. From Table 4, as the Nb/C ratio increases, the amount of NbC precipitated in the matrix also increases, which is basically consistent with the research results of Zhou et al. [28]. The mass fraction of NbC precipitates in the steel with an Nb/C ratio of 13 is 0.404 wt%. Substituting this value into Equation (5), the volume fraction of the precipitate particles is approximately 0.404 wt%. Using Equation (4), the yield strength increment $\Delta \sigma_{p}$ is calculated to be 207.9 MPa. Following the same procedure, the yield strength increment for the steel with Nb/C = 9 is calculated to be 136.9 MPa. These results demonstrate that the precipitation-strengthening effect of NbC phases increases with the Nb/C ratio.

## 5. Conclusions

In this study, the influence of the Nb/C ratio on the mechanical properties of B50A789G steel was investigated through mechanical property testing. The mechanisms of Nb in the steel were analyzed using microstructural characterization and quantitative calculations. The following conclusions were drawn:

1. At the same tempering time, increasing the Nb/C ratio enhances the strength and hardness of B50A789G steel but reduces its toughness. Notably, the impact energy decreases significantly when the Nb/C ratio reaches 13. With prolonged tempering time, the tensile strength of B50A789G steel gradually decreases. For steels with Nb/C ratios of 7 and 9, the tensile strength exhibits a continuous decline. However, when the Nb/C ratio is increased to 11 and 13, the decline in tensile strength is delayed.
2. Increasing the Nb/C ratio leads to a higher volume fraction of nanoscale NbC precipitates, which enhances their pinning effect on dislocations. This retards the widening of martensitic laths and delays the onset of lath decomposition. Simultaneously, the amount of coarse primary NbC precipitates in the steel also increases with the Nb/C ratio.
3. For steels with Nb/C ≤ 11, the prior austenite grain size gradually refines as the Nb/C ratio increases. During this stage, the improvement in material strength is primarily attributed to the combined effects of grain refinement strengthening and precipitation strengthening provided by Nb. However, when the Nb/C ratio is further increased to 13, the grain refinement strengthening effect of Nb becomes less significant, and the increase in strength is mainly driven by the precipitation strengthening effect of a higher volume fraction of nanoscale NbC precipitates.
4. The results show that increasing the Nb/C ratio in B50A789G steel can effectively improve the strength of the steel. However, a high Nb/C ratio will cause a large amount of precipitation of the primary NbC phase in the steel, resulting in a significant decrease in the impact energy. For practical applications, further refinement studies on Nb and C contents should be conducted according to specific requirements to determine the optimal Nb/C ratio.

## Figures and Tables

**Table 1:** Chemical composition of the tested steel (wt%).

Nb/C	C	Mn	Si	Cr	Ni	Mo	Cu	Nb	V	N	Fe
	0.03–0.05	0.3–0.8	0.2–0.5	14.0–16.0	6.2–6.8	0.6–1.0	1.35–1.75	0.3–0.6	≤0.10	≤0.030	Bal.
7	0.046	0.48	0.43	14.43	6.5	0.83	1.58	0.32	0.059	0.028	Bal.
9	0.044	0.48	0.43	14.43	6.5	0.84	1.6	0.39	0.062	0.028	Bal.
11	0.044	0.48	0.42	14.28	6.52	0.84	1.58	0.5	0.058	0.031	Bal.
13	0.045	0.47	0.43	14.51	6.48	0.83	1.56	0.58	0.058	0.031	Bal.

**Table 2:** Nb solid solution content in austenite at 1038 °C.

Nb/C	7	9	11	13
Content of Nb in steel (wt%)	0.32	0.39	0.5	0.58
Solid solution of Nb in steel (wt%)	0.09	0.14	0.29	0.28

**Table 3:** Thermo-Calc calculation result statistics.

Nb/C	7	9	11	13
Initial full austenite temperature (°C)	1327	1317	1316	1295
Primary NbC precipitation amount (wt%)	0	0.05	0.15	0.23

**Table 4:** Mass fractions of NbC phase and yield strength increments for steels with different Nb/C ratios.

Nb/C	Mass Fractions of NbC (wt%)	Yield Strength Increments (MPa)
9	0.306	136.9
13	0.404	207.9

## Data Availability

The original contributions presented in the study are included in the article, further inquiries can be directed to the corresponding authors.
